# Soft Sensing of Non-Newtonian Fluid Flow in Open Venturi Channel Using an Array of Ultrasonic Level Sensors—AI Models and Their Validations

**DOI:** 10.3390/s17112458

**Published:** 2017-10-26

**Authors:** Khim Chhantyal, Håkon Viumdal, Saba Mylvaganam

**Affiliations:** Faculty of Technology, Natural Sciences, and Maritime Sciences, University College of Southeast Norway, Kjølnes Ring 56, 3918 Porsgrunn, Norway; hakon.viumdal@usn.no (H.V.); saba.mylvaganam@usn.no (S.M.)

**Keywords:** soft sensing in open channels, non-Newtonian flow, ultrasonic scanning of open channel flow, neural networks, Bayesian regularization learning, fuzzy logic, support vector regression

## Abstract

In oil and gas and geothermal installations, open channels followed by sieves for removal of drill cuttings, are used to monitor the quality and quantity of the drilling fluids. Drilling fluid flow rate is difficult to measure due to the varying flow conditions (e.g., wavy, turbulent and irregular) and the presence of drilling cuttings and gas bubbles. Inclusion of a Venturi section in the open channel and an array of ultrasonic level sensors above it at locations in the vicinity of and above the Venturi constriction gives the varying levels of the drilling fluid in the channel. The time series of the levels from this array of ultrasonic level sensors are used to estimate the drilling fluid flow rate, which is compared with Coriolis meter measurements. Fuzzy logic, neural networks and support vector regression algorithms applied to the data from temporal and spatial ultrasonic level measurements of the drilling fluid in the open channel give estimates of its flow rate with sufficient reliability, repeatability and uncertainty, providing a novel soft sensing of an important process variable. Simulations, cross-validations and experimental results show that feedforward neural networks with the Bayesian regularization learning algorithm provide the best flow rate estimates. Finally, the benefits of using this soft sensing technique combined with Venturi constriction in open channels are discussed.

## 1. Introduction

One of the important phases in extracting oil and gas is drilling from the surface down to the reservoir. Due to high temperature and pressure conditions in the bottom-hole, there is a high risk of failure while drilling. Drilling fluid circulation plays a vital role in safe and efficient drilling operations. The drilling fluid can be water-based or oil-based depending on the type of reservoir. While drilling, the drilling fluid is continuously pumped down into the wellbore through the drill pipe. The circulating drilling fluid returns to the surface through the annulus, i.e., the space between the drill pipe and the wellbore. The drilling fluid circulation continues until the desired depth is reached. The primary functions of drilling fluid circulation are stabilizing the wellbore, the cleaning borehole and transporting rock cuttings. These functions are dependent on the properties of drilling fluid, among which density, viscosity and flow rate are the most important ones. The viscosity and other rheological properties of circulating fluid regulate the hole cleaning and transportation of rock cuttings [1].

In the context of this paper, variations of viscosity are not taken into account. The drilling fluid density is responsible for wellbore stability. For any reservoir, there exists a certain pressure window where the drilling operation can be performed safely. The pressure window extends from formation pressure ($P_{f}$) to formation fracture pressure ($P_{ff}$). The wellbore pressure must be maintained within this pressure window ($P_{ff}-P_{f}$) for safe drilling. In the case of reservoir failure, two main problems might occur. If the wellbore or bottom-hole pressure ($P_{b}$) is greater than formation pressure ($P_{f}$), the high-pressure drilling fluid displaces the formation fluids and enters into the formation pores, causing a fluid loss. If the drilling fluid pressure is greater than formation fracture pressure ($P_{ff}$), it fractures the formation, and the fluid loss further increases, which is a state of lost circulation while drilling. Alternatively, if ($P_{b}<P_{f}$), the high-pressure formation fluids and gasses displace the drilling fluid, which is the state of kick while drilling. The kick should be detected as early as possible, as it can initially lead to wellbore stability problems, and in the extreme case, it might result in the blowout of the whole rig, e.g., the Deepwater Horizon explosion [2]. The bottom-hole pressure depends on the hydrostatic pressure exerted by the circulating drilling fluid, choke pressure and frictional pressure. The hydrostatic pressure is mainly responsible for bottom-hole pressure, which is dependent on the density of drilling fluid or drilling fluid weight. In this way, by monitoring the density of circulating drilling fluid, the wellbore pressure can be maintained within the acceptable pressure window [1].

Loss of the drilling fluid, kick, unexpected changes in surge pressure and any uncontrolled high flow rates of drilling fluid should be indicated to the operator (human or autonomous) by a timely and preventive alarm, so that the operator takes the necessary actions to limit material damages and hazards to personnel. The early detection of these problems can lead to less fluid loss, less formation damage, lower drilling costs and, above all, increased safety with minimized maintenance costs. One of the simplest methods for early detection is the so-called delta flow method, which utilize the difference between inflow and outflow measurements in a circulation loop. To implement the delta flow method, two flow measurements for drilling fluid entering the well (inflow) and drilling fluid returning from the well (outflow) are needed. When the inflow exceeds outflow, lost circulation in the loop is a possibility. On the other hand, for inflow less than outflow, the possible occurrence of kick is indicated. Other different methods for kick and lost circulation detection are discussed in [3,4,5,6].

Therefore, the aim is to accurately determine the delta flow in the circulation loop. There are different types of flow measurement systems for delta flow measurement in the literature [7,8,9,10,11]. To point out some of them, the conventional pump strokes counter, rotatory pump speed counter and Coriolis mass flowmeter can be used for inflow measurement and the standard paddle meter, ultrasonic level meter, a prototype rolling float meter and open channel Venturi flow meter can be used for outflow measurements. With some adjustments, the magnetic flow meter and Doppler ultrasonic flow meter can be used for both inflow and outflow measurements, although due to high attenuation of ultrasonic signals in drilling fluids, this might not be a suitable option. The Coriolis mass flowmeter delivers one of the smallest uncertainties in flow metering. It has a very high accuracy with both Newtonian and non-Newtonian fluids. However, bubbles and mechanical vibrations affect the Coriolis measurement [12]. Therefore, it is not appropriate to use for outflow measurement, where the returning fluid contains rock cuttings, formation gasses and formation liquids. In addition, the Coriolis meter is an expensive option. Different flowmeters based on reliability and accuracy are discussed in [11]. The analysis concludes that the magnetic flowmeter or Doppler ultrasonic flowmeter can be used for inflow measurement, and prototype rolling float meters can be used for outflow measurement. Speers and Gerhrig [8] have presented the usage of magnetic flowmeters for delta flow measurement. However, magnetic flowmeters are limited to water-based or conductive drilling fluids. Another problem with magnetic flowmeters is the the requirement of a U-tube designed pipe to ensure a complete filled pipe. With this desgin, there will be a settlement of rock cuttings in the U-tube when the flow velocity is low. In this paper, the usage of an open channel with Venturi constriction is presented where the limitations using the magnetic flowmeter no longer exist [9,10].

In an open channel with Venturi constriction, the upstream pressure relative to the level in the control section is used to estimate the flow rate of the fluid [13]. Fluids flow from the subcritical to supercritical flow condition due to the Venturi effect [14]. The critical depth is determined within the control section, and the level of the fluid in the upstream is measured. Ultrasonic or radar sensors can be used for level measurement, which can be used to estimate the flow of the fluid through the open channel [15].

To study the possibility of using Venturi constriction in an open channel for flow measurement, a flow loop is available at University College of Southeast Norway (USN), Campus Kjølnes, Norway. As a part of this study, the Computational Fluid Dynamics (CFD) simulation study is investigated in [16,17]. The possibility of using the Saint Venant equation for non-Newtonian fluid through the open channel is presented in [18]. The usage of the Ensemble Kalman Filter (EnKF) for estimating non-Newtonian fluid flow in an open channel is studied in [19]. This mathematical approach presented in [18,19] is computationally demanding and is only applicable to a slow system with a large sampling time. These considerations indicate that for real-time monitoring and controlling purposes, these approaches are not suitable. In [20], static Artificial Neural Network (ANN) and Support Vector Regression (SVR) techniques are implemented for flow measurement in an open channel. The simulation-based study shows that both static ANN and SVR models have more than a 100-times faster response time as compared to the mechanistic model presented in [18,19]. With an assumption of delta flow measurement as a dynamic problem, dynamic ANN with different learning algorithms is investigated in [21]. Further, the Bernoulli equation can be implemented for the flow rate estimation. The fundamental Bernoulli equation for the flow of an incompressible fluid in an inclined channel takes the following form:

(1) $$\frac{P_{1}}{\rho g}+\frac{u_{1}^{2}}{2g}+z_{1}=\frac{P_{2}}{\rho g}+\frac{u_{2}^{2}}{2g}+z_{2}$$

where *P*, *u*, *z*, ρ and *g* are fluid pressure, fluid velocity, elevation of the channel relative to the datum, fluid density and acceleration due to gravity, respectively, with the subscripts indicating two distinct positions in the inclined channel. The further simplification of Equation (1) along with continuity equation, $u_{1}\times A_{1}=u_{2}\times A_{2}$ gives Equation (2),

(2) $$Q_{v}=A_{1}A_{2}{\left[2g\left\{\frac{\left(h_{2}-h_{1}\right)+\left(z_{2}-z_{1}\right)}{A_{2}^{2}-A_{1}^{2}}\right\}\right]}^{1\;/\;2}$$

where $Q_{v}$, $h_{1}$, $h_{2}$, $A_{1}$ and $A_{2}$ are volumetric flow rate, upstream level measurement, level measurement at the throat, area before the constriction and area at the constriction, respectively. The mass flow rate $\left(Q_{m}\right)$ of the fluid can be calculated as $Q_{m}=Q_{v}\times \rho$.

In theory, the simplified equation (Equation (2)) can be used to estimate the flow rate using a set of spatial samplings of the open surface of the fluid in the Venturi channel, leading to a set of level measurements. However, due to non-ideal conditions (for example: compressible fluid, sediments leading to variations of the cross-sections, fluctuations of the open surface of the non-Newtonian fluid, varying velocity profile in the cross-section of the channel, etc.) and uncertainties in the geometrical parameters (for example: cross-sectional area of the fluid in the channel, channel elevation, etc.), we are resorting to a soft sensor approach using non-invasive measurements in this work. Hence, the present paper focuses on using different empirical methods such as fuzzy logic, ANN and Support Vector Machine (SVM) with both simulation and experimental results.

The system description is presented in Section 3, and different proposed methods are described in Section 4. Finally, the results from simulations and experimental studies are presented in Section 5 and Section 6, respectively.

## 2. Requirements for a Drilling Fluid Flowmeter

In an earlier paper [9], addressing the need for reliable and accurate flow measurement of non-Newtonian fluids, the following features are expected from a suitable flowmeter:

- Over the full range of flow, the reliability and accuracy of measurements are guaranteed.
- In the common drilling operational environment, an accuracy of 1.5–3 L/s for flow rates up to 75 L/s.
- For any type of drilling fluids (water and oil based) in the viscosity range 1–200 cP and density range of 1000–2160 kg/m${}^{3}$, the accuracy should be maintained.

The methods presented here may be used for non-intrusive measurements of drilling fluids in many sectors satisfying all these requirements. Although some changes in these expected features may be seen in the practices of different operators, these can be used as design guidelines.

## 3. System Description

**Figure 1:**
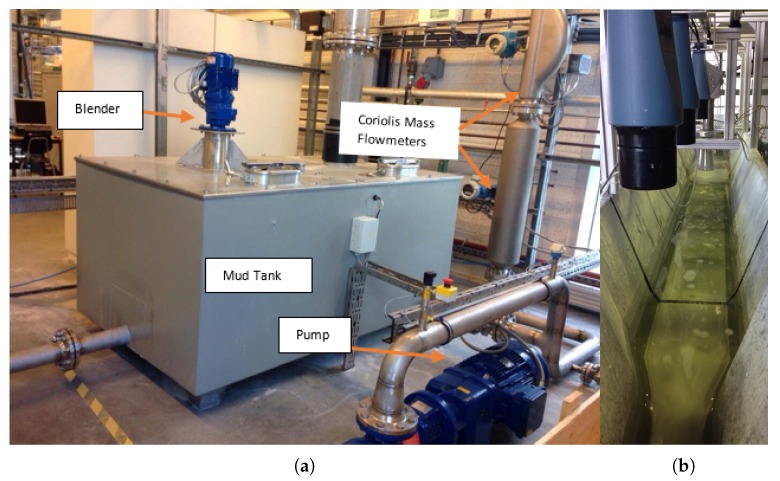
(**a**) Test flow loop at University College of Southeast Norway, Kjølnes Campus, showing mud tank, blender, pump and Coriolis flowmeters. (**b**) Open Venturi channel with ultrasonic level sensors.

Figure 1 shows a flow loop available at USN consisting of a mud tank and a blender for mixing. Different model-drilling fluids are available for testing purposes. The centrifugal pump is used to pump the model-drilling fluid from the mud tank through the pipelines to the open channel with Venturi constriction as shown in Figure 1b. The pumped fluid flows through the open channel and down to the mud tank forming a complete flow loop. The flow loop includes different types of measurement systems like the pressure transmitter, temperature transmitter, Coriolis mass flowmeters, Gamma sensor dedicated for density measurement, differential pressure sensor, an open channel with Venturi constriction, an inclination sensor and different ultrasonic level sensors.

**Figure 2:**
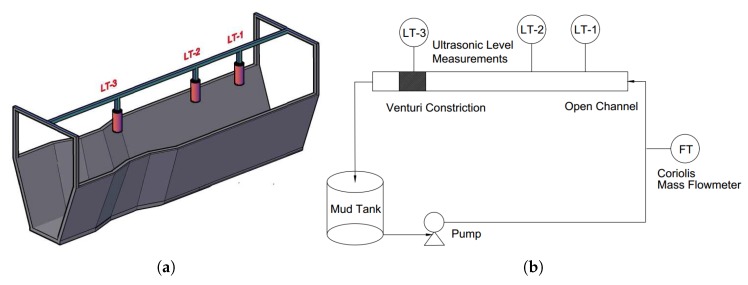
(**a**) An open channel with Venturi constriction and three ultrasonic level sensors (LT-1, LT-2, and LT-3). (**b**) Extremely simplified P&IDfor the flow loop with the measurands used in the study. The schematic shows the “hard sensors” in the system under study. The focus is on drilling fluid (also called “mud”) mass balance based on flow measurements [21].

In this study, an accurate Coriolis mass flowmeter is used as a reference meter for all comparisons of results from empirical models. The open channel has a trapezoidal cross-section with Venturi constriction. The upstream length is long enough to ensure fully developed flow before entering the constriction. Further, the channel can be inclined to the horizontal at different angles to analyze different flow conditions. Three different ultrasonic level sensors are installed over the open channel, giving levels of fluid in the channel, which will be used for flow measurements, as discussed in the following sections. Figure 2a shows the 3D view of open channel with Venturi constriction and three ultrasonic level sensors. The schematic of the system is given in Figure 2b.

For the current study, a model-drilling fluid consisting of potassium carbonate (as the densifier) and xanthan gum (as the viscosifier) is used. The fluid is viscoplastic in nature with a density of 1153 kg/m${}^{3}$, and its viscosity values are within 23–180 cP for corresponding shear rates within 500–1 s${}^{-1}$.

This water-based non-Newtonian fluid with the properties given above is used in assessing the performance of a method of estimating its volumetric flow by sampling the levels of the open surface of the fluid flowing in the Venturi channel with an array of non-invasive ultrasonic level sensors. The performance of this soft sensing of the flow rate should satisfy the criteria outlined in Section 2.

**Figure 3:**
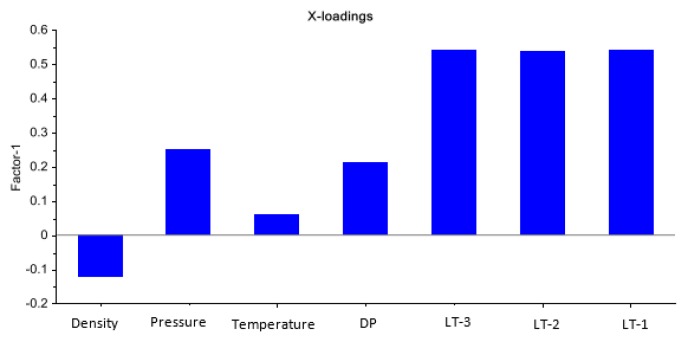
Loading weight plot using the Partial Least Square (PLS) method to identify the most important variables correlated with mass flow measurements. The three level measurements show obviously high PLS scoring [20].

The first step in conceiving of a suitable empirical model is the identification of suitable input feature space for estimating the mass flow rate of a drilling fluid. The Partial Least Square (PLS) method used in steady state conditions from earlier studies [20] shows that two upstream level measurements, LT-1 and LT-2, and the level measurement at the throat, LT-3, are highly correlated with Coriolis mass flow measurement, as shown in the loading weights plot in Figure 3. The list of different measurement devices with the respective technical specifications considered for the identification of input and output features for empirical models is presented in Table 1.

**Figure 4:**
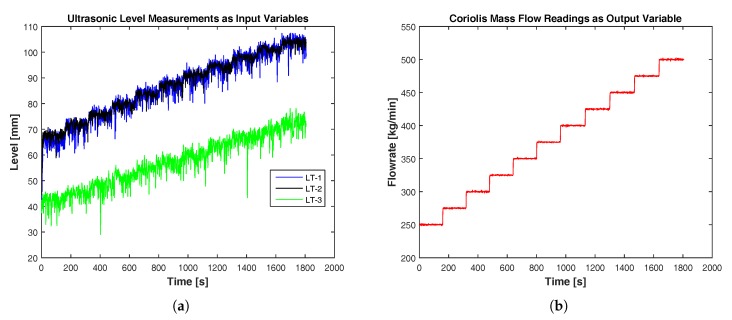
Input and output variables used in flow rate models. (**a**) Three ultrasonic level measurements, namely LT-1, LT-2, and LT-3, as inputs. (**b**) Flow measurement using the Coriolis mass flowmeter as the reference output [21].

For developing models, about 1800 data samples are used for each of the three input variables (ultrasonic levels) and the single output variable (Coriolis flow rate). The samples are obtained at the data sampling rate of one sample per second using compactDAQ in the LabVIEW environment. The ranges, units and input/output types of each variable considered for modeling are tabulated in Table 2. The simultaneous inputs and output measurements are shown in Figure 4. In Figure 4a, the level measurements LT-1 and LT-2 are measuring almost the same upstream levels. LT-1 measures comparatively lower levels, which is due to the energy losses during the backward flow of the fluid initiated by the hydraulic jump near the constriction. The level measurements are noisy due to the presence of foams in the flowing fluid and due to random uncertainties in ultrasonic measurements. The data samples are normalized in the range of 0–1. From the 1800 normalized data samples, 75%, 12.5% and 12.5% of the data are used for training, validation and testing purposes, respectively.

## 4. Methods Used with Selected Algorithms

In this work, different Artificial Intelligence (AI) methods are used to estimate the flow rate of the non-Newtonian fluid. Under this section, AI methods like Fuzzy Logic (FL), feedforward and feedback ANN and Support Vector Regression (SVR) are briefly discussed.

### 4.1. Fuzzy Logic Approach

**Figure 5:**
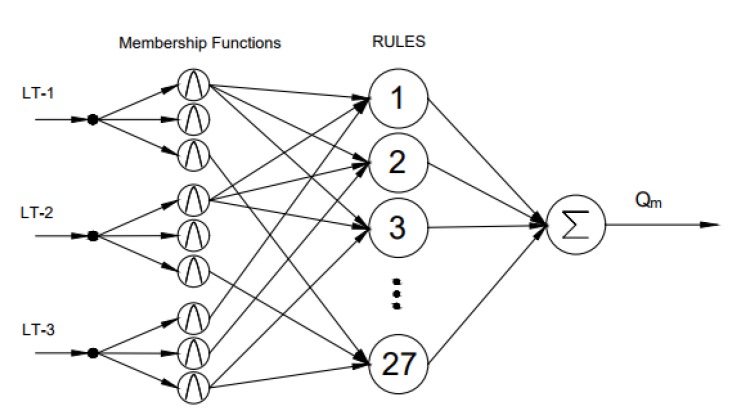
A Sugeno-type Fuzzy Logic architecture with outputs from “hard” sensors LT-1, LT-2 and LT-3 as crisp inputs and drilling fluid outflow as the crisp soft sensor output. Adapted from [22] and modified.

Fuzzy Logic (FL) is an approach where the computing is based on degrees of truth rather than crisp true or false values. The FL tool can be considered as a function that receives inputs and gives an output based on the defined rules and membership functions. Analysis of the literature [23,24,25,26] shows that the fuzzy logic approach can be successfully applied for learning, predicting and controlling. Figure 5 shows the architecture of the Sugeno-type fuzzy logic with ANFIS used in predicting mass flow rates based on three ultrasonic level measurements. In this work, the Sugeno-type fuzzy logic with the Adaptive Neuro-Fuzzy Inference System (ANFIS) is used.

### 4.2. Feedforward Artificial Neural Network

**Figure 6:**
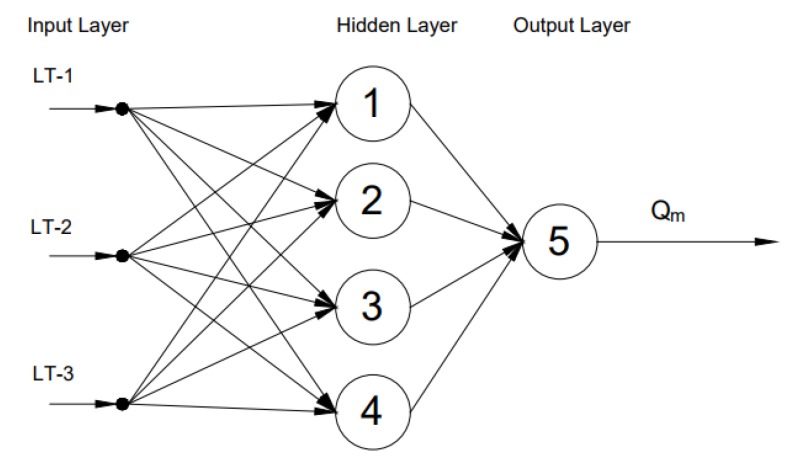
A feedforward artificial neural network architecture with an input layer, hidden layer and an output layer. Three ultrasonic level measurements, LT-1, LT-2 and LT-3, are inputs to the network and drilling fluid outflow as the soft sensor output from the network [20].

ANN is a kind of non-linear mapping system suitable for pattern recognition, regression problems, image compression, etc. [27,28,29,30,31,32,33]. In the network, the bias of the neuron and weights between the neurons are the model parameters. These model parameters are tuned based on a certain cost function using a suitable learning algorithm [27,28]. A feedforward ANN is a static ANN that uses current inputs to estimate current outputs. The architecture of the feedforward ANN always moves in one direction as shown in Figure 6.

In this paper, feedforward ANN with three different learning algorithms, Levenberg–Marquardt (LM) learning, Bayesian Regularization (BR) learning and Scaled Conjugate Gradient (SCG) learning, are investigated. The cost function for LM learning and SCG learning algorithms is the mean squared error defined by Equation (3), and the generalization is performed using the early stop technique. Both of these algorithms are faster in learning. However, regarding memory, LM learning takes more memory compared to SCG learning [34]; whereas, the BR learning algorithm involves the minimization of mean squared error and weight parameters of a network. The cost function for BR learning is defined by Equation (4), and the generalization is performed using regularization [34].

(3) $$\begin{array}{ccc} J(w,b) & = & \frac{1}{2n}\sum_{i=1}^{n}{\left(∥p_{i}-T_{i}∥\right)}^{2} \end{array}$$

(4) $$\begin{array}{ccc} J(w,b,\lambda ) & = & \frac{1}{2n}\sum_{i=1}^{n}\left\{{\left(∥p_{i}-T_{i}∥\right)}^{2}+\lambda W^{2}\right\} \end{array}$$

where *J* represents the cost function, which is a function of weights (*w*) and bias (*b*). Parameters *n*, *p* and *T* represent the number of samples, model prediction and target value, respectively. *W* is the weight parameter vector and λ the regularization parameter or weight decaying factor.

### 4.3. Feedback Artificial Neural Network

**Figure 7:**
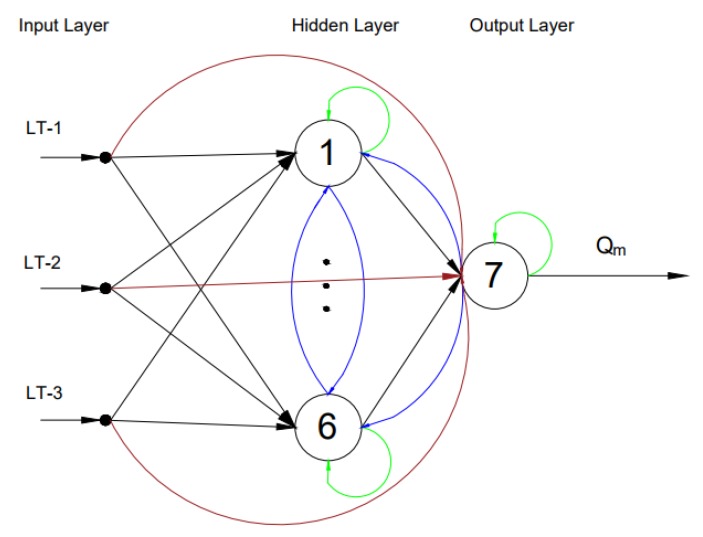
The architecture for feedback ANN with self-feedback (denoted by green connections), feedback loops (denoted by blue connections) and direct connections from inputs to the output neuron (denoted by brown connections). Ultrasonic level measurements as input vectors to the network and the drilling fluid flow rate as the output from the network [21].

A feedback ANN is a dynamic ANN that uses previous inputs and outputs to estimate current outputs. The architecture of a fully-connected feedback ANN consisting of feedback loops and self-feedback loops is shown in Figure 7.

In this paper, feedback ANN with three different learning algorithms, Back Propagation Through Time (BPTT), Real-Time Recurrent Learning (RTRL) and Extended Kalman Filter Learning (EKF), is studied. BPTT is an extension of the classical gradient-based back-propagation algorithm where the feedback ANN architecture is unfolded into feedforward ANN with a different number of folds [35,36,37,38]. It converges faster, but it is an offline learning algorithm [35,36,37,38]. On the other hand, both RTRL and EKF are online learning algorithm. RTRL is simple and the slowest converging algorithm, whereas EKF is complex and the fastest learning algorithm [35,36,37,38]. Mean squared error defined by Equation (3) is used as a cost function in all three feedback learning algorithms.

### 4.4. Support Vector Regression

**Figure 8:**
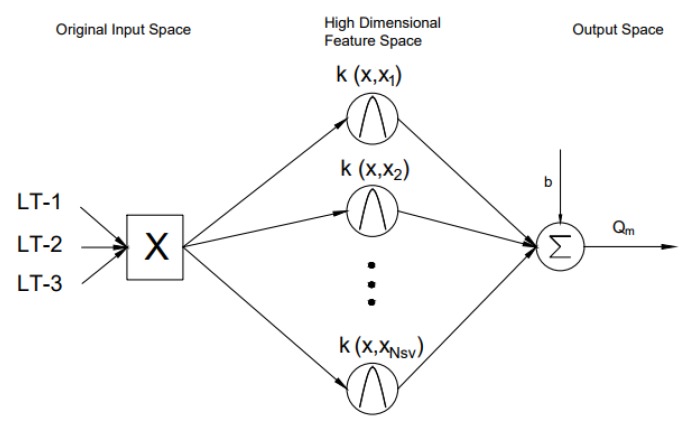
An architecture of Support Vector Regression (SVR) showing a mapping from input space to high dimensional feature space using the radial basis kernel function. Ultrasonic level measurements as input vectors and the drilling fluid flow rate as the output [20].

The Support Vector Machine (SVM) technique is applied in applications like classification problems, pattern recognition, time series predictions and regression problems [39,40,41,42]. The basic idea of the SVM technique is to perform a mapping of original data in the input space into the higher dimensional feature space through non-linear mapping functions. In this paper, SVM is used in its regression form, defined by (Equation (5)) as Support Vector Regression (SVR) [39].

(5) $$y=\sum_{i=1}^{N_{SV}}\left(w_{i}\cdot \phi_{i}\left(x\right)\right)+b$$

where ϕ(x) (also represented as $k(x,x_{i})$, *k* representing the kernel function) is the mapping function from the input space to the feature space, *b* is the bias term, *x* represents the input, *y* represents the output and $N_{SV}$ is the number of support vectors. The architecture of SVR used in this paper is shown in Figure 8. Three ultrasonic level measurements (X) are transformed into higher dimensional feature space using the Radial Basis Function (RBF) kernel. Thus, the obtained higher dimensional feature is mapped with mass flow rate to develop a regression model.

### 4.5. Building AI Models

**Figure 9:**
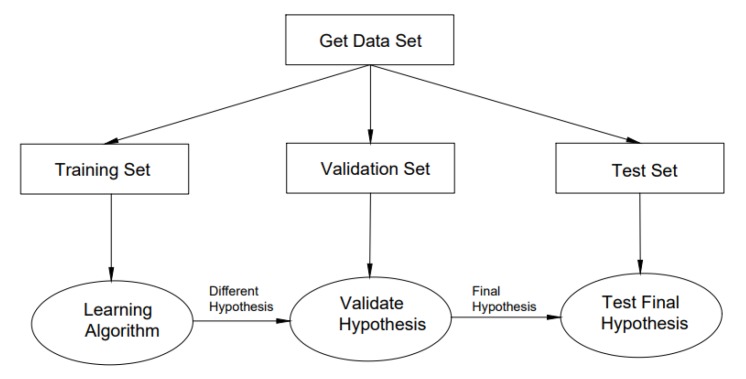
A flowchart for training, validating and testing different AI techniques.

Different AI models are developed using the data from three ultrasonic level measurements LT-1, LT-2 and LT-3 as inputs and Coriolis mass flow readings as the output. The dataset is normalized and divided into three sets for training, validation and testing. Different empirical relations (hypothesis) between inputs and output are developed using the training dataset. The empirical models developed are then validated leading to the final hypothesis with associated optimal model parameters. Finally, the eight models are tested for their performance. The flowchart for training, validating and testing all the AI models is shown in Figure 9. A pseudocode for training, validating and testing different AI models is presented below.

(a) % Get and normalize dataset
  dataSet=GetDataSet()
  data=Normalize(dataSet)
(b) % Divide dataset into training, validation and testing sets
  trainingData=FindTrainingSet(data)
  validationData=FindValidationSet(data)
  testData=FindTestSet(data)
(c) % Construct two arrays of different AI techniques and corresponding learning algorithms
  $artificialIntelligenceTechniques={\{}^{\prime }fuzzyLogicAlgorithm^{\prime },{\;}^{\prime }feedforwardLMAlgorithm^{\prime },\;$
  ${}^{\prime }feedforwardBRAlgorithm^{\prime },{\;}^{\prime }feedforwardSCGAlgorithm^{\prime },{\;}^{\prime }feedbackBPTTAlgorithm^{\prime },\;$
  ${}^{\prime }feedbackRTRLAlgorithm^{\prime },{\;}^{\prime }feedbackEKFAlgorithm^{\prime },{\;}^{\prime }svrAlgorithm^{\prime }\}$
  $LearningAlgorithms={\{}^{\prime }ANFISLearningAlgorithm^{\prime },{\;}^{\prime }LMLearningAlgorithm^{\prime },\;$
  ${}^{\prime }BRLearningAlgorithm^{\prime },{\;}^{\prime }SCGLearningAlgorithm^{\prime },{\;}^{\prime }BPTTLearningAlgorithm^{\prime },\;$
  ${}^{\prime }RTRLLearningAlgorithm^{\prime },{\;}^{\prime }EKFLearningAlgorithm^{\prime },{\;}^{\prime }SVRLearningAlgorithm^{\prime }\}$
(d) % Train different AI techniques using training data set
  FORalgorithm= 1–8
  artificialIntelligenceTechniques{algorithm}=LearningAlgorithms{algorithm}(trainingData)
  ENDFOR
(e) % Validate all the AI techniques using the validation dataset
  FORalgorithm= 1–8
  Validate(artificialIntelligenceTechniques{algorithm},validationData)
  ENDFOR
(f) % Test all the AI techniques using test set
  FORalgorithm= 1–8
  Test(artificialIntelligenceTechniques{algorithm},testData)
  ENDFOR

### 4.6. Cross-Validation for Model Selection

In this work, the cross-validation technique is used for model selection. For the purpose of model selection, the dataset is divided into *k* number of folds (k=10, in our case). Out of *k* subsets, the (k−1) set is used for training or calibrating the model, and remaining subsets are used for validating or testing the model. The process is repeated by changing the validation subset, and then, the average cross-validation error is calculated. The model with the lowest cross-validation error is considered to be the best model using this technique [43,44].

Further, Table 3 shows the pros and cons of different AI methods used in this study. The selection of a suitable model is application dependent.

## 5. Simulation Study

Based on the setup discussed in Section 3, the results of the simulation study are presented under this section. As discussed in Section 3, we have measurements from three level measurements from ultrasonic sensors LT-1, LT-2 and LT-3 and the Coriolis mass flowmeter. All the models are evaluated using Mean Absolute Percentage Error (MAPE) and coefficient of determination $R^{2}$. The low value of MAPE represents the better performance of the model, as it gives the error percentage value. On the other hand, the value of $R^{2}$ closer to 1.0 indicates that the model predictions and target values are highly correlated. The parameter tuning of models is one of the most important steps in empirical modeling. In this paper, the parameters of ANN models are tuned based on the grid search method followed by some adjustment using trial and error. Most of the parameters of the Sugeno-type fuzzy logic model are tuned automatically, and the rest of the parameters are based on trial and error. Optimal selection of SVR model parameters is made using the process described in [45]. Table 4 shows the optimal parameters used in all the models. All the symbols used in Table 4 are given in Appendix A.

**Figure 10:**
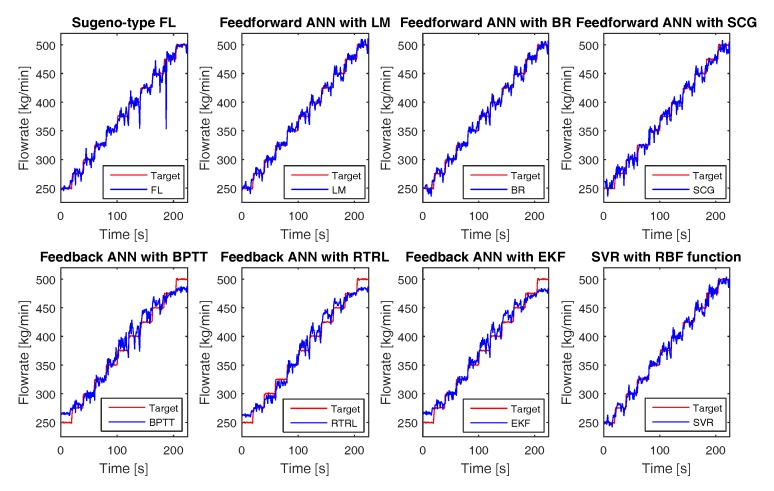
The flow rate estimates of non-Newtonian fluid based on simulations compared to the flow rate using from Coriolis meter. Both in static and dynamic conditions, simulation results and Coriolis meter readings tally very well.

Figure 10 shows the flow rate estimations of non-Newtonian fluid using all the proposed empirical models compared to the Coriolis mass flow measurements. From these simulation studies, it can be seen that all the proposed models can track the changes in flow rates with high accuracy and are capable of describing both the steady state and dynamic behaviors of the fluid flow.

Table 5 shows the comparison of the results from different proposed models based on MAPE and $R^{2}$. Based on these performance criteria, feedforward ANN with Bayesian Regularization and Levenberg–Marquardt learning algorithms are the best models to be implemented with the lowest percentage error and highest correlation with target values. However, other proposed models also have very accurate predictions.

For further analysis, four different types of models are selected, one from each method. The cross-validation technique with 10-folds is implemented in each of the selected models. Table 6 shows the selected models with corresponding cross-validation error. Based on the cross-validation check, the best model for flow rate estimation is feedforward ANN with the Bayesian regularization model, which has the lowest cross-validation error. It is due to the fact that the BR learning algorithm uses regularization for the generalization of a model. The regularization parameter prevents the model from being over-fit by minimizing the connection weights.

## 6. Experimental Study

**Figure 11:**
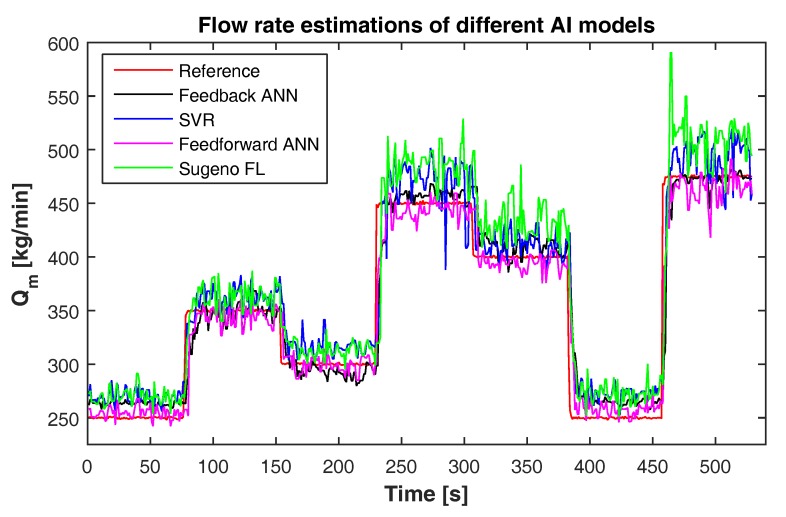
The flow rate estimates using feedback ANN with the RTRL learning algorithm, SVR with the RBF kernel function, feedforward ANN with the Bayesian regularization learning algorithm and Sugeno-type FL compared to the Coriolis meter readings.

Based on the simulation study, four different models, the Sugeno-type fuzzy logic model, feedforward ANN with BR learning model, feedback ANN with RTRL learning and SVR with RBF kernel model, are implemented in the flow loop. Figure 11 shows the experimental results obtained with non-Newtonian fluid using these models. During the experiments, the set point is randomly varied between 250 and 475 kg/min. In response, all the models can track the varying references with good accuracy. Table 7 shows the comparison of the experimental performance of different models based on MAPE, $R^{2}$ and Root Mean Squared Error (RMSE). From the performance table, it can be seen that the feedforward ANN with BR learning model having the lowest MAPE and RMSE of 3.28% and 0.3 L/s respectively, and the highest $R^{2}$ of 94% is the best generalized model for estimating the flow rate of the non-Newtonian fluid. However, all these models give much smaller RMSE with acceptable uncertainties for a flowmeter needed for the current application.

**Figure 12:**
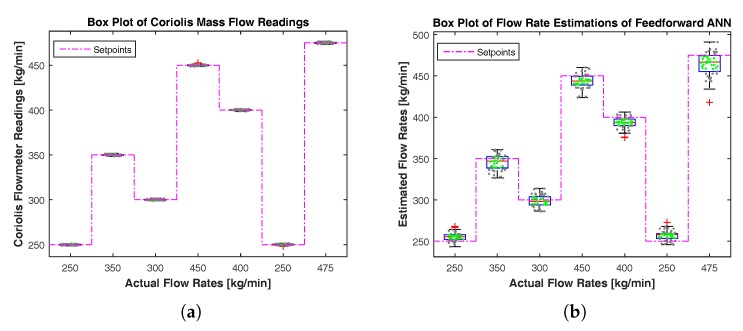
Box plots showing the spread of the sensor measurements and model-based estimates at different flow rates. Green dots, black dots and the red plus sign represent measurements/estimates within the Interquartile Range (IQR), within the upper and lower bounds, but out of IQR, and outliers, respectively. (**a**) Box plots for Coriolis mass flow meter readings. (**b**) Box plots for flow rate estimations of feedforward ANN.

Figure 12 shows box plots for Coriolis flowmeter readings and feedforward ANN estimates at different flow rates. As a reference, a varying setpoint is also included in the plots. In the box plot, a blue box is an Interquartile Range (IQR), and the central red line is a median of measurements/estimates. Two whiskers above and below the box are 1.5×IQR from the edge of the box, which corresponds to the 99.3% confidence interval for a normal distribution. Hence, the size of a box represents the spread or variance of measurements/estimates. Figure 12a shows that the sizes of boxes for Coriolis readings are very small, and the medians are very close to the reference line. This represents the high accuracy of the Coriolis flowmeter; whereas, the size of boxes for the estimates of feedforward ANN are comparatively larger, as shown in Figure 12b. The sizes of boxes are small at low flow rates and large at high flow rates, representing low and high variances, respectively. In addition, the medians for feedforward ANN are slightly displaced from the reference line showing some limited accuracy in estimations.

**Figure 13:**
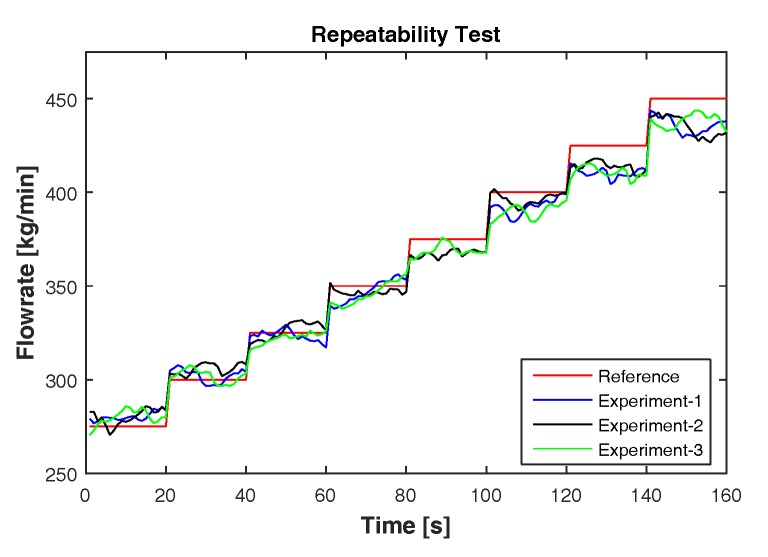
Repeatability test conducted on three different experiments under the same conditions. The estimated flow rates using feedforward ANN in different experiments are compared against the reference setpoints.

Further, feedforward ANN is considered under the repeatability test as shown in Figure 13. Under similar conditions, three experiments are performed, and the estimates of feedforward ANN are compared. For the comparison, only the steady state measurements are considered. Table 8 shows the results of the repeatability test. The calculated MAPE and $R^{2}$ show that the estimates of feedforward ANN are highly repeatable.

**Figure 14:**
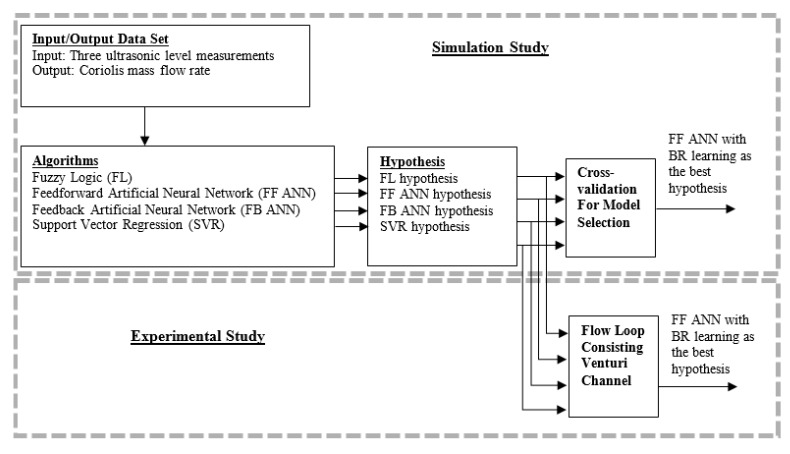
Overview of the strategies used during the simulation and experimental studies. Feedforward with Bayesian Regularization (BR) learning comes out as the best approach for soft sensing of the flow rate.

The simulation and experimental study is summarized in Figure 14.

## 7. Conclusions

The drilling operation is one of the main phases of extracting oil and gas from the reservoir in oil and gas industries. In the context of geothermal applications, it helps to reach the necessary depth for achieving the high-temperature environment for heat transfer. In the context of oil and gas boring operations, due to extreme conditions in the bottom-hole, there is a high risk of failure while drilling. In unusual cases, there might be two problems while drilling: the influx of formation fluid (i.e., kick) and loss of circulation fluid. One of the best ways to detect these problems is the delta flow method, which utilizes the difference in inflow and outflow measurements of drilling fluid in a flow loop. There are different methods to perform accurate inflow measurements discussed in the literature. However, it is complicated to measure the outflow measurement accurately, particularly so for non-Newtonian fluids. In this paper, we introduce different empirical models and present both simulation and experimental results based on the comparison to readings from the Coriolis mass flowmeter. The starting point for this particular investigation is the set of three ultrasonic height measurements. The question is whether we can estimate the bulk flow velocity based only on these three parameters using non-invasive techniques. This is where soft sensor models come into play. The results from extensive experiments with non-Newtonian model-drilling fluids in the research laboratory of Statoil and in the flow loop at USN are used to develop the soft sensor models presented in this paper.

Different empirical models presented in this work are: the Sugeno-type fuzzy logic model, feedforward ANN models with three learning algorithms (Levenberg–Marquardt learning, Bayesian regularization learning and scaled conjugate gradient learning), feedback ANN models with three learning algorithms (back propagation through time, real-time recurrent learning and extended Kalman filter learning) and support vector regression model with the radial basis function as the kernel function. For these models, the partial least square method is used to identify the inputs and output variables. In the simulation study, feedforward ANN with LM learning and BR learning are found to be the best models based on the MAPE and $R^{2}$. Further, some of the models are considered under the 10-fold cross-validation technique for suitable model selection. In this study, feedforward ANN with BR learning is selected to be the best generalized model with the lowest cross-validation error. Similar to simulation results, the flow rate estimates using feedforward ANN with BR learning are close to the results from the experiments. However, all the presented models are capable of tracking both the static and dynamic behavior of time-varying non-Newtonian fluid flow. The results presented here along with the measurements based on the array of ultrasonic transducers confirm that the flow rate of the drilling fluids could be measured satisfying the requirements specified in [9].

For future work, the quality and quantity of the training and validating datasets can be improved. As the proposed modeling is mainly dependent on the type of data, we believe that improvement in data measurement and extraction will improve the performance of the models. For this purpose, the first step will be filtering the noise from the data and performing other signal processing techniques to improve the signal information.

The technique presented here paves the way for realizing a simple and effective soft sensing system for monitoring a commonly-occurring module in the fossil fuel and renewable industries, viz. the operational unit for transport, cleaning recovery and mass balance budgeting of a costly and environmentally-hazardous drilling fluid, which is non-Newtonian. The soft sensing of the fluid flow rate using an array of non-intrusive and non-invasive ultrasonic transducers could spare the operators expensive maintenance costs and improve autonomous operation of plants in conventional fossil fuel and emerging renewable energy industries. For interested researchers, the data used in this study are made available in the web portal of this journal.

## Figures and Tables

**Table 1:** Technical specifications of different measurement devices considered for initial identification of input and output features for empirical models. Based on information from the vendors.

Measurement Devices	Vendor	Model	Range	Uncertainty
Coriolis meter (flow rate)	Endress + Hauser	Promass 63F	0–1000 (L/min)	±0.10%
Coriolis meter (density)	Endress + Hauser	Promass 63F	0.8–1.8 (g/cc)	±0.001
Temperature transmitter	Endress + Hauser	TI00110REN14	−50–200 (${}^{\circ }$C)	±0.19
Ultrasonic level sensor	Rosemount	3107	0.3–12 (m)	±0.25%
Pressure transmitter	Aplisens	PCE-28 Smart	0–7 (bar)	±0.10%
Differential pressure	Aplisens	APRE-2000	0–250 (mbar)	±0.10%

**Table 2:** Input and output variables used in flow rate models with the units, ranges and variable types.

Variables	Range	Units	Type
LT-1	37.2–107.5	mm	Input
LT-2	28.9–78.3	mm	Input
LT-3	44.3–106.6	mm	Input
Coriolis mass flow rate	250–500	kg/min	Output

**Table 3:** Pros and cons of different AI methods used in this study.

AI Methods	Pros	Cons
Fuzzy Logic	Simple to implement and can be a good alternative for solving complex problems.	The performance depends on the model parameters and rules. Insufficient knowledge about the system can degrade the performance.
Artificial Neural Network	Suitable for modeling non-linear problems and one of the best choices for a large number of input features.	Training is computationally expensive.
Support Vector Regression	Works very well with non-linear problems and is not biased by outliers.	The algorithm is more complex and is not the best method for a large number of features.

**Table 4:** The optimal parameters used in all the proposed models for estimating the flow rate of the non-Newtonian fluid. All models implemented off-line using MATLAB. Model parameters, mostly software specific, are described in the nomenclature.

Methods	Optimal Parameters
Sugeno-type fuzzy logic	$N_{m}=3$, $N_{r}=27$, $M_{m}$ = Gaussian-type, output = linear-type
Feedforward ANN with LM learning	$N_{h}=1$, $N_{n}=4$, α=0.1, Epoch=1000
Feedforward ANN with BR learning	$N_{h}=1$, $N_{n}=4$, α=0.1, Epoch=1000
Feedforward ANN with SCG learning	$N_{h}=1$, $N_{n}=4$, α=0.1, Epoch=1000
Feedback ANN with BPTT learning	$N_{n}=7$, α=0.1, $N_{f}=7$, Epoch=200, $N_{i}=1$, $N_{o}=3$
Feedback ANN with RTRL learning	$N_{n}=7$, α=0.1, Epoch=200, $N_{i}=4$, $N_{o}=4$
Feedback ANN with EKF learning	$N_{n}=7$, α=0.1, Epoch=200, $N_{i}=4$, $N_{o}=4$
Support vector regression with RBF	C=500, ϵ=0.01, σ=0.1

**Table 5:** The comparison of the simulation performance in estimating output flow; all the proposed models are based on MAPE or $R^{2}$. Selected methods (represented by bold numbers) are considered in cross-validation for model selection.

Methods	MAPE (%)	$R^{2}$
Sugeno-type fuzzy logic	1.74	0.98
Feedforward ANN with LM learning	1.58	0.99
Feedforward ANN with BR learning	1.58	0.99
Feedforward ANN with SCG learning	1.97	0.99
Feedback ANN with BPTT learning	2.89	0.97
Feedback ANN with RTRL learning	2.57	0.98
Feedback ANN with EKF learning	2.71	0.98
Support vector regression with RBF	1.61	0.99

**Table 6:** The model selection using the cross-validation technique.

Methods	Cross-Validation Error (%)
Sugeno-type fuzzy logic	1.89
Feedforward ANN with BR learning	1.59
Feedback ANN with RTRL learning	2.70
Support vector regression with RBF	1.75

**Table 7:** The comparison of the experimental performance of different models used for estimating flow based on MAPE, $R^{2}$ and RMSE.

Methods	MAPE (%)	$R^{2}$	RMSE (kg/min)	RMSE (L/s)
Sugeno-type fuzzy logic	7.72	0.83	34.94	0.51
Feedforward ANN with BR learning	3.28	0.94	20.90	0.30
Feedback ANN with RTRL learning	4.25	0.91	25.02	0.36
Support vector regression with RBF	6.43	0.89	28.30	0.41

**Table 8:** Repeatability test performed with three experiments under the same conditions. The results are evaluated based on MAPE and $R^{2}$.

Experiments	MAPE (%)	$R^{2}$
1	1.97	0.975
2	1.90	0.977
3	2.01	0.975

## Supplementary Materials

The Supplementary Materials are available online at http://www.mdpi.com/1424-8220/17/11/2458/s1.

## Appendix A. List of Symbols and Abbreviations

Appendix A consist of a list of symbols and abbreviations used in this work.

**Symbols**		**Abbreviations**	
*b*	Bias term	AI	Artificial Intelligence
*C*	Punishing factor	ANFIS	Adaptive Neuro-Fuzzy Inference System
Epoch	Maximum epochs for learning	ANN	Artificial Neural Network
*g*	Acceleration of gravity	BPTT	Back Propagation Through Time
*h*	Fluid level	BR	Bayesian Regularization
*J*	Cost function	CFD	Computational Fluid Dynamics
$M_{m}$	Type of membership function	DP	Differential Pressure
*n*	Number of samples	EKF	Extended Kalman Filter
$N_{f}$	Number of folding	FB	Feedback
$N_{h}$	Number of hidden layers	FF	Feedforward
$N_{i}$	Number of previous inputs	FL	Fuzzy Logic
$N_{m}$	Number of membership function	IQR	Interquartile Range
$N_{n}$	Number of hidden neurons	LM	Levenberg–Marquardt
$N_{o}$	Number of previous outputs	LT	Level Transmitter
$N_{r}$	Number of rules	MAPE	Mean Absolute Percentage Error
$N_{sv}$	Number of support vectors	PLS	Partial Least Square
*p*	Model predictions	RBF	Radial Basis Function
*P*	Fluid pressure	RMSE	Root Mean Squared Error
$P_{b}$	Formation pressure	RTRL	Real-Time Recurrent Learning
$P_{ff}$	Formation fracture pressure	SCG	Scaled Conjugate Gradient
$Q_{m}$	Mass flow rate	SVM	Support Vector Machine
$Q_{v}$	Volumetric flow rate	SVR	Support Vector Regression
$R^{2}$	Coefficient of determination	USN	University College of Southeast Norway
*T*	Target		
*u*	Fluid velocity		
*w*	Weight		
*W*	Weight parameter vector		
*X*	Input matrix		
*Y*	Outputs		
*z*	Elevation relative to a datum		
α	Learning rate		
ϵ	Tolerance zone		
ρ	Fluid density		
σ	Width of RBF function		
λ	Regularization parameter		
ϕ(·)	Mapping function		
