# Supplementary material for: Soft Sensing of Non-Newtonian Fluid Flow in Open Venturi Channel Using an Array of Ultrasonic Level Sensors—AI Models and Their Validations
Source: Sensors (Basel). 2017 Oct 26;17(11):2458. doi: 10.3390/s17112458 (PMC5713661; doi:10.3390/s17112458)
Supplement: Supplementary file 1 [file sensors-17-02458-s001.zip › Highlights/Highlights.docx]

Highlights:

- There is a dire need for accurate flow rate measurement of drilling fluids for stable, safe and efficient drilling operations.
- This work presents different empirical models (Fuzzy Logic approach, Artificial Neural Network approach, and Support Vector Machine approach) to perform non-intrusive and non-invasive estimations of drilling fluid flow.
- With simulations, feedforward neural networks with Levenberg Marquardt (LM) and Bayesian Regularization (BR) learning algorithms gave the best performance based on Mean Absolute Percentage Error and Squared Correlation Coefficient criterions.
- 10-fold cross-validation technique and extensive experimental analysis show that the feedforward neural network with BR learning is the best model for estimating the drilling fluid flow.
- The results presented in this work along with the measurements based on the array of ultrasonic transducers confirm that the flow rate of the drilling fluids could be measured satisfying the key requirements of the industries involved.
