# Supplementary figures and images for: Soft Sensing of Non-Newtonian Fluid Flow in Open Venturi Channel Using an Array of Ultrasonic Level Sensors—AI Models and Their Validations
Source: Sensors (Basel). 2017 Oct 26;17(11):2458. doi: 10.3390/s17112458 (PMC5713661; doi:10.3390/s17112458)

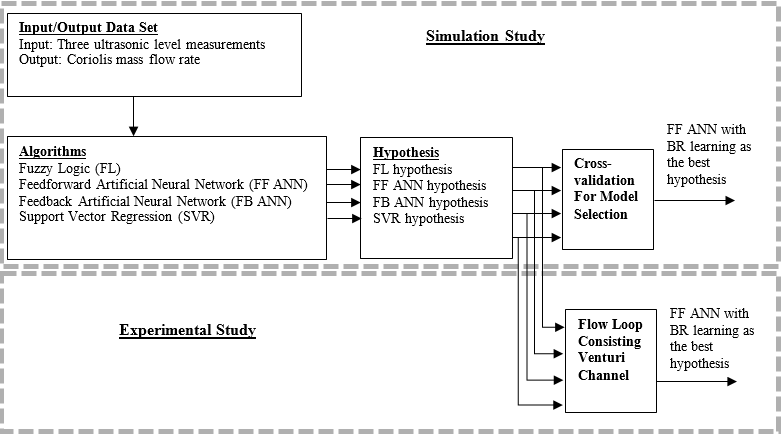

Supplement: Supplementary file 1 [file sensors-17-02458-s001.zip › Figures/Figure_14.PNG]

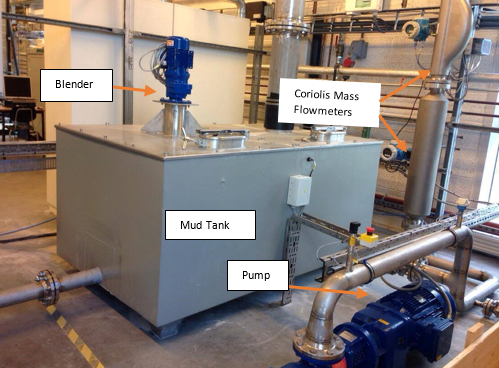

Supplement: Supplementary file 1 [file sensors-17-02458-s001.zip › Figures/Figure_1a.png]

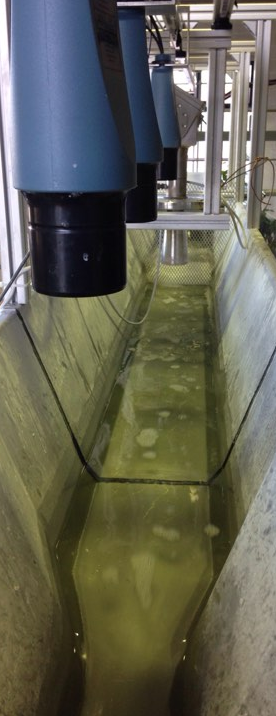

Supplement: Supplementary file 1 [file sensors-17-02458-s001.zip › Figures/Figure_1b.PNG]

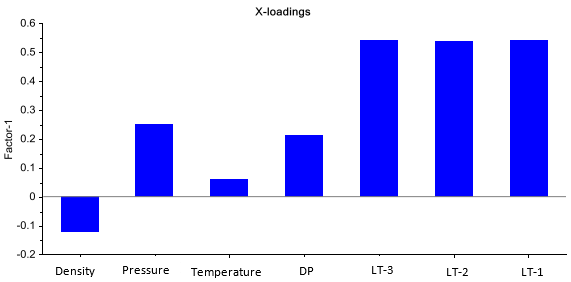

Supplement: Supplementary file 1 [file sensors-17-02458-s001.zip › Figures/Figure_3.png]

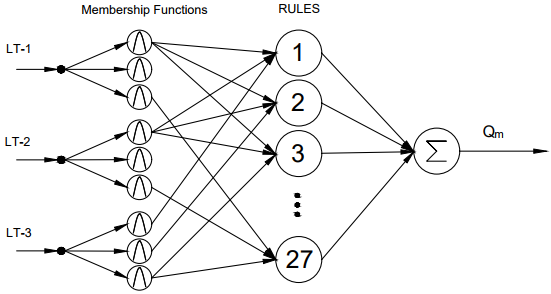

Supplement: Supplementary file 1 [file sensors-17-02458-s001.zip › Figures/Figure_5.PNG]

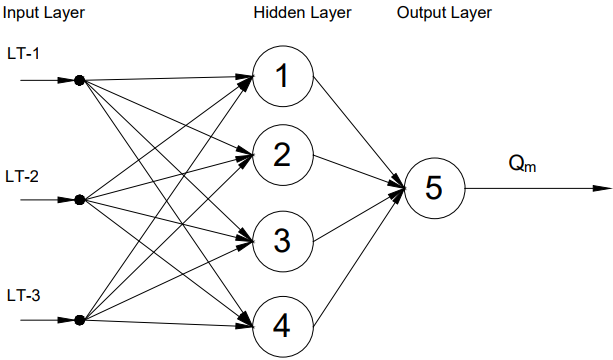

Supplement: Supplementary file 1 [file sensors-17-02458-s001.zip › Figures/Figure_6.PNG]

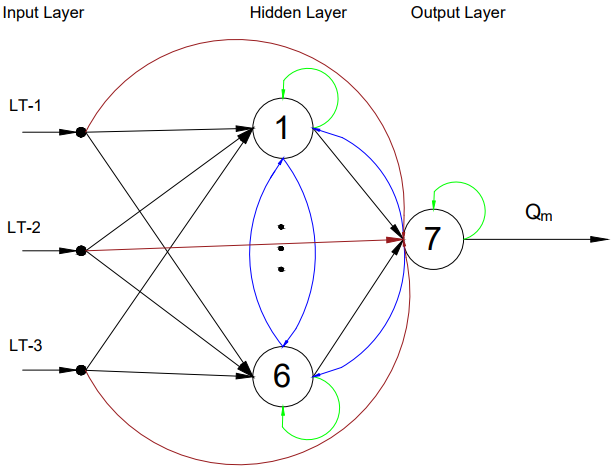

Supplement: Supplementary file 1 [file sensors-17-02458-s001.zip › Figures/Figure_7.PNG]

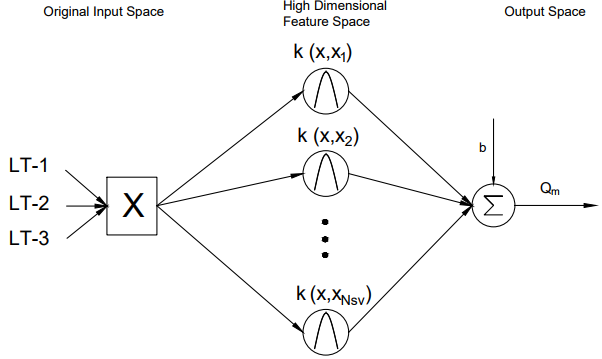

Supplement: Supplementary file 1 [file sensors-17-02458-s001.zip › Figures/Figure_8.PNG]

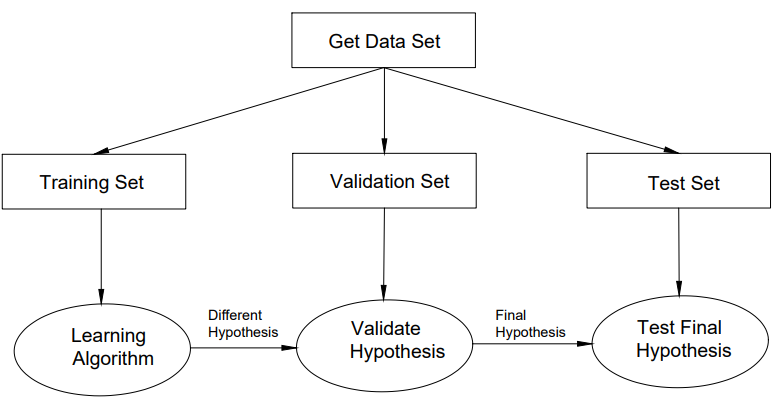

Supplement: Supplementary file 1 [file sensors-17-02458-s001.zip › Figures/Figure_9.PNG]
